# Prevent: what is pre-criminal space?

**DOI:** 10.1192/pb.bp.116.054585

**Published:** 2017-08

**Authors:** David Goldberg, Sushrut Jadhav, Tarek Younis

**Affiliations:** 1 University College London, London, UK; 2 Université du Québec à Montréal, Montreal, Canada

## Abstract

Prevent is a UK-wide programme within the government's anti-terrorism strategy aimed at stopping individuals from supporting or taking part in terrorist activities. NHS England's Prevent Training and Competencies Framework requires health professionals to understand the concept of pre-criminal space. This article examines pre-criminal space, a new term which refers to a period of time during which a person is referred to a specific Prevent-related safeguarding panel, Channel. It is unclear what the concept of pre-criminal space adds to the Prevent programme. The term should be either clarified or removed from the Framework.

The Prevent Training and Competencies Framework^1^ begins thus:

‘Prevent is part of the Government's counter-terrorism strategy CONTEST and aims to stop people becoming terrorists or supporting terrorism; as such it is described as the only long term solution to the threat we face from terrorism. Prevent focuses on all forms of terrorism and operates in a pre-criminal space, providing support and redirection to vulnerable individuals at risk of being groomed in to terrorist activity before any crimes are committed. Radicalisation is comparable to other forms of exploitation; it is therefore a safeguarding issue staff working in the health sector must be aware of. [… ] Staff must be able to recognise signs of radicalisation and be confident in referring individuals who can then receive support in the pre-criminal space.’ (p. 5)

The Framework is cascaded down the National Health Service (NHS) hierarchies in England to ensure that all front-line staff in the NHS receive mandatory training in the Prevent process. NHS staff refer patients considered vulnerable to radicalisation to local Prevent leads and onward to Prevent case managers and the Channel panel. The Channel acts as a multi-agency panel along the lines of other safeguarding panels in England.

This Training and Competency Framework follows from the *Prevent Duty Guidance*,^2^ the *Channel Duty Guidance*^3^ and the *Channel: Vulnerability Assessment Framework*^4^ produced by the government following *CONTEST: The United Kingdom's Strategy for Countering Terrorism*^5^ and the Counter-Terrorism and Security Act 2015. The Framework does not name these documents but mentions two other documents which focus on multi-agency working. The first, *Safeguarding Children and Young People*,^6^ details the roles and competencies for healthcare staff when working with other professionals to safeguard children and young people. The second, *Building Partnerships, Staying Safe*,^7^ stresses the importance of effective interprofessional working.

The focus of the Framework is on training. Among the competencies listed within is to understand the concept of pre-criminal space. This article examines this term using publicly accessible government documents and internet searches performed on Google and Google Scholar. It is a textual analysis, read in the context of other government documents,^8^ which presumes that what is read may not necessarily be what the authors initially intended. Meanings applied to words, particularly new compound words, gain significance over time and use.^9^ One of the presumptions of this method is that textual analysis may not relate directly to what is happening in practice.

## Pre-criminal space in the NHS England Framework

‘Pre-criminal space’ appears four times in the Framework document, including twice in the introduction:

1. ‘Prevent focuses on all forms of terrorism and operates in a pre-criminal space’ (p. 5)
2. ‘… individuals [ … ] can then receive support in the pre-criminal space’ (p. 5)
3. ‘… aware[ness that] [ … ] the health sector contribution operates in pre-criminal space’ (p. 8)
4. ‘Understand [ … ] the concept of pre-criminal space’ (p. 10).

The meaning of ‘pre-criminal space’ can be deduced from these quotes. ‘Prevent focuses on all forms of terrorism and operates in a pre-criminal space’ suggests that pre-criminal space may relate to specific physical spaces and times where professionals ‘operate’ or act within the aims of the Prevent programme. The statement that ‘individuals [ … ] can then receive support in the pre-criminal space’ suggests that the professional activity involved is ‘support’. What constitutes ‘support’ is detailed in the *Channel Duty Guidance*.^3^ The fact that ‘the health sector contribution operates in pre-criminal space’ implies that other health sector activities may not operate in the pre-criminal space. In what way NHS activity in this ‘space’ is different from that in other space is not stated anywhere. The need for health professionals to understand ‘the concept of pre-criminal space’ suggests that there is an important difference between what pre-criminal space is and what it is not. Read in the context of Prevent^2^ and *Channel Duty Guidance*,^3^ pre-criminal space is likely to start on acceptance of a referral of a person within the Channel panel, or perhaps on referral or discussion of the possibility of referral by NHS staff to Channel personnel. Thus, pre-criminal space has temporal and spatial aspects. As the time and space is decided by negotiation with and between professionals, the term also has inter-professional dimensions. This is supported by Framework naming documents that focus on effective multidisciplinary work rather than the Prevent and Channel Duty guidelines.^6,7^

The Framework document views the Prevent programme as part of the wider safeguarding agenda. However, there is one important difference compared with other safeguarding panels: the coordinator of the Channel process, the Channel Police Practitioner (CPP), is a police officer or is employed by the police (ref. 3, para. 30). Thus, unlike other safeguarding procedures, the police have a central coordinating function.

## Coining of ‘pre-criminal space’

The term pre-criminal space is new. It was introduced by this Framework and cascaded to all trusts by NHS England. The term does not occur in non-NHS Prevent documents or in CONTEST. References to pre-criminal space since the term was introduced are largely found in trust documentation and NHS PowerPoint presentations, together with some journalist reports and blogs. For instance, the *Telegraph* comments on the police use of the term and possible repercussions on state-Muslim relations.^10^

All NHS trusts in England are mandated to enact the Framework document and produce policy or guidance documents. None of these documents define pre-criminal space any further. Many use pre-criminal space with quotation marks, a few prefacing the term with ‘so-called’. The usual statement is a reiteration of ‘Prevent operates in the pre-criminal space’. Occasionally the hyphen is replaced by a space between ‘pre’ and ‘criminal’ but the words are never placed directly together. The most detailed definition we have found comes from a glossary in a Prevent document from Mersey Care NHS Trust, beginning with a precautionary note: ‘These definitions relate to PREVENT and are not always authoritative in any wider context.’ ‘Pre-Criminal Activity/Space’ is explained by focusing on ‘multi-agency working to ensure that individuals are diverted away before any crime is committed’.^11^ This definition merges ‘space’ with multi-agency activity.

## Denotation and connotation

So far we have argued that pre-criminal space refers to the time when a person is engaged by the Channel panel and related professionals. It denotes the time, space and interprofessional activity involved in planning, coordinating support and possibly monitoring in the NHS England Prevent programme. What is unclear is whether the Channel process and panel meetings are in any way different from other multi-agency activities. This may be deduced by examining possible connotations of the term based on participants' understanding of language use in its social context.

Pre-criminal space consists of three terms: ‘pre’ meaning before, ‘criminal’ as a person who has committed a crime or repeated crimes, and ‘space’ as a continuous physical area. ‘Pre’ appears to modify the second term, ‘criminal’, rather than space. Hyphens are not usually used after prefixes such as ‘pre’, unless the resulting meaning becomes ambiguous, for example ‘pre-order’ rather than ‘preorder’. Thus, the use of the hyphen both gives a separation between pre-crime and crime and creates the link. The term implies that the ‘space’ is pre-criminal, not the individual. While the use of the term ‘space’ suggests a physical space, such as a meeting room, there are no references to where the vulnerable person is to be supported. ‘Space’ in this context is used as a relational concept, common in expressions such as ‘I need space to think’, meaning ‘I need a place for myself, away from certain social relationships’.

## The derivation of pre-criminal space

The etymology of the term is significant to the discussion. ‘Pre-crime’ and ‘space’, as separate words, suggest they are potentially independent concepts. The term ‘precrime’ was said to be coined by science fiction writer Philip K. Dick in his short story *Minority Report*.^12^ The drama is based on the concept that crime has not occurred yet but will occur in future unless measures are taken. The belief that crime can be prevented by identification and intervention has a long history. In the 19th century, Lambroso's theory of criminal atavism famously purported to identify future criminals by their abnormal physical appearance. In recent years, criminologists use the term pre-crime to criticise the move to criminalise people prior to the committing of crime.^13^ More recently, the term ‘pre-criminal space’ has been used in the US security industry in relation to Islamic fundamentalist terrorism.^14^ We find no evidence that the concept of ‘space’ in pre-criminal space derives from academic theorising about space. Depending on context, however, space connotes elements of time as well as physical or abstract forms of space. Space in pre-criminal space can thus gain different metaphoric associations depending on the immediate social context. Pre-criminal space may describe the physical space where ‘support’ is planned, ‘operated’ or monitored. It may also relate to the time that the person is subject to the Channel panel process, or to differences in the social rules of interprofessional interaction, as compared with different safeguarding panel discussions. Finally, it may refer to differing social identities of the person referred to the Channel panel and the professionals involved.

## Rhetoric

The Framework provides an abbreviated and reasoned argument in support of the Prevent programme and the involvement of health professionals. Like all arguments, it persuades through the use of the metaphoric qualities which words gain in use. There are two sets of metaphors within the Framework, one pertinent to NHS professionals and the other to the police. The health metaphors are borrowed from structural engineering, with vulnerability and support suggesting the diathesis-stress model. The person is weakened from external assault or internal deficiency; structural support is provided from outside so the individual can withstand potential assaults or threats. Even the WRAP acronym (Workshops to Raise Awareness of Prevent) suggests physical bodily protection against external threat. The criminal justice terms (i.e. radicalisation, extremism, CONTEST, counter-terrorism, strategy) suggest a heightened spatial metaphor, polarisation and direction of movement. The creation of the term pre-criminal space may be an attempt to bridge the health and police use of metaphor: the health metaphor aiming for stability and predictability, while the criminal justice metaphor focuses on adaptability. The lack of effective definition of pre-criminal space allows for evolving inter-agency norms during the Channel process to vary with context.

If new concepts emerge by visual analogy,^15^ pre-criminal space can be seen as an extension of crime prevention. It can be visualised as a system of continuous and contained passages. Prior to the vent (derived from the Latin root, as in *prevent),* professionals direct radicals, people who have an attractive or repulsive charge or energy, past the vent and into the channel. As the radicals pass along the vent their charge or energy becomes less strong and the radical no longer moves towards the extreme.

If the term pre-criminal space mobilises healthcare professionals and Channel partners towards a crime prevention role of health intervention, pre-criminal space has the potential to act as a form of ‘excitable speech’ to alert listeners to the threat of terrorism.^16^ The rhetorical use of the term pre-criminal space during the Channel process could be perceived as an attempt to persuade professionals to disclose information or make decisions they would otherwise not make in other safeguarding forums and in comparable circumstances.

## Opinion

The Training and Competency Framework is an action plan, laying out who does what, when and with whom. For the implementation of Prevent, terms need to be defined to such a degree that the participants can understand the concepts involved. Indeed, the Framework itself asks NHS staff to understand the concept of pre-criminal space (p. 10). Pre-criminal space clearly denotes a period of time in the Prevent process without adding further meaning and keeping open the opportunity for the term's possible rhetorical use in Channel meetings.

One synonym for pre-criminal space is crime prevention. This raises the possibility of changing the wording of the Framework to replace this obscure and newly coined term with ‘crime prevention’. Alternatively, discussion and clarification of the concept of pre-criminal space would allow it to define the participants' relationships as the crime prevention activity proceeds. Further multidisciplinaiy research linking NHS documents to practice may enable the Prevent guidance and framework to be linked with practice. When the time comes for the Prevent framework to be updated (although no such date is given in the document), we recommend that the term should be clarified or removed.
